# Weighted gene co-expression network analysis and whole genome sequencing identify potential lung cancer biomarkers

**DOI:** 10.3389/fonc.2024.1355527

**Published:** 2024-05-24

**Authors:** Mireguli Abudereheman, Zhengjun Lian, Baidurula Ainitu

**Affiliations:** Oncology Department, The Eighth Affiliated Hospital of XinJiang Medical University, Urumqi, China

**Keywords:** lung cancer, tuberculosis, co-expression, whole exome sequencing, EGFR

## Abstract

**Background:**

Tuberculosis (TB) leads to an increased risk of lung cancer (LC). However, the carcinogenetic mechanism of TB remains unclear. We constructed gene co-expression networks and carried out whole-exome sequencing (WES) to identify key modules, hub genes, and the most recurrently mutated genes involved in the pathogenesis of TB-associated LC.

**Methods:**

The data used in this study were obtained from the Gene Expression Omnibus (GEO) and WES. First, we screened LC-related genes in GSE43458 and TB-related genes in GSE83456 by weighted gene co-expression network analysis (WGCNA). Subsequently, we screened differentially expressed genes related to LC and TB in GSE42834. We also performed WES of 15 patients (TB, n = 5; LC, n = 5; TB+LC, n = 5), constructed mutational profiles, and identified differences in the profiles of the three groups for further investigation.

**Results:**

We identified 278 hub genes associated with tumorigenesis of pulmonary TB. Moreover, WES identified 112 somatic mutations in 25 genes in the 15 patients. Finally, four common genes (EGFR, HSPA2, CECR2, and LAMA3) were confirmed in a Venn diagram of the 278 hub genes and the mutated genes from WES. KEGG analysis revealed various pathway changes. The PI3K–AKT signaling pathway was the most enriched pathway, and all four genes are included in this pathway. Thus, these four genes and the PI3K–AKT signaling pathway may play important roles in LC.

**Conclusion:**

Several potential genes and pathways related to TB-associated LC were identified, including EGFR and three target genes not found in previous studies. These genes are related to cell proliferation, colony formation, migration, and invasion, and provide a direction for future research into the mechanisms of LC co-occurring with TB. The PI3K–AKT signaling pathway was also identified as a potential key pathway involved in LC development.

## Introduction

Lung cancer (LC) is a leading cause of cancer-related deaths and a critical barrier to increasing life expectancy worldwide (1). Tuberculosis (TB) is one of the major deadly infectious diseases and remains a global public health threat (2). TB increases the risk of LC and affects the prognosis of LC patients. The incidence of lung cancers is approximately 11-fold higher in the cohort of patients with TB compared with non-tuberculosis subjects (26.3 versus 2.41 per 10,000 person-years) (3, 4). Several prospective and retrospective studies have suggested that TB is associated with an increased risk of lung cancer (5–7).

Early diagnosis bias and late treatment strategies for TB might be factors responsible for the high co-occurrence of TB and LC (8–11). TB diagnosis is performed by QuantiFERON-TB Gold In-Tube tests as a gold standard (5, 12). Pulmonary comorbidities can considerably obscure LC symptoms and delay diagnosis, or may preclude a comprehensive diagnostic examination with adequate illness staging. The risk of LC should be assessed before starting treatment for TB, with the aim of preventing LC development. Therefore, there is an urgent need to explore signature genes closely associated with the development of LC to allow early diagnosis of the development of LC in TB patients.

Co-expression networks are useful to describe pairwise relationships between gene transcripts (13). Here, we used weighted gene co-expression network analysis (WGCNA) to calculate associations between gene significance (GS) and module membership (MM), analyze the correlation between modules to construct a weighted gene co-expression network, and merge differentially expressed genes (DEGs) with key module genes for functional analysis. By constructing the protein–protein interaction (PPI) network, we detected certain hub genes as key factors regulating the occurrence of LC.

For further research, 15 patients (TB, n = 5; LC, n = 5 (3 adenocarcinoma, 2 non-small cell lung cancer); TB+LC, n = 5) were recruited and whole-exome sequencing (WES) was performed on the primary fresh tissues. Mutational profiles of the 15 patients were constructed based on the sequencing data, and differences in the mutational profiles between the three groups of patients were investigated further. The combination of the WGCNA and the identified DEGs revealed four target hub genes (EGFR, HSPA2, CECR2, and LAMA3) that may be potential biomarkers for LC diagnosis and treatment.

## Material and methods

### Data information

The GSE43458, GSE83456, and GSE42834 datasets were obtained from NCBI Gene Expression Omnibus (GEO) ( https://www.ncbi.nlm.nih.gov/geo/ ). GSE43458 consists of 80 lung cancer samples and 30 control samples run on an Affymetrix Human Gene 1.0 ST Array [HuGene-1_0-st]. GSE83456 contains 45 pulmonary tuberculosis samples and 61 control samples run on an Illumina HumanHT-12 V4.0 Expression BeadChip. GSE42834 contains 20 controls, eight patients with lung cancer, and 19 pulmonary tuberculosis patients, also run on an Illumina HumanHT-12 V4.0 Expression BeadChip. The R packages affy and annotate were used to process the raw data, make an expression matrix, and match probes to their gene symbol. The R package Limma was used to screen the DEGs based on the GSE42834 data. All DEGs were screened with q-value < 0.001 and |log2FC| > 0.5 as thresholds. The common differential genes in these results were selected for functional analysis.

### Patient samples

This study was performed according to the Declaration of Helsinki (2013) of the World Medical Association. The study protocol was approved by the Ethics Committees of The Xinjiang Uygur Autonomous Region Chest Hospital (approval number 2021BAT011). Fresh primary tissues were collected from 15 pathologically confirmed patients undergoing surgery for lung cancer, pulmonary tuberculosis, or lung cancer combined with pulmonary tuberculosis at The Eighth Affiliated Hospital of XinJiang Medical University (Urumqi, China). Histological diagnosis of tumors was performed and confirmed by two pathologists. Samples were immediately frozen in liquid nitrogen and stored at −80°C until further analysis. The clinicopathological features of the 15 patients are presented in **Supplementary Table S5.**

### WGCNA construction

Based on the expression and clinical pathological data of the GSE43458 and GSE83456 datasets, the genes showing the top 60% variance were selected for weighted gene co-expression network analysis (WGCNA). This was used to calculate the correlation coefficients between genes for clustering and to construct a co-expression weighted network. The hierarchical clustering function was used to classify genes with similar expression profiles into modules based on the topological overlap matrix (TOM) dissimilarity with a minimum size of 30 for the gene dendrogram. The blue modules were significantly associated with TB, and the turquoise and yellow modules were significantly associated with LC. The dissimilarity of module eigengenes (MEs) was calculated to choose a cut-off to merge some modules. Lastly, the network of eigengenes was also visualized.

### Identification of clinically significant modules

To determine the appropriate modules, we computed the association between MEs and clinical traits. The log10 transformation of the P-value (GS = lgP) in the linear regression between gene expression and clinical data was then used to establish gene significance (GS). The average GS for every gene in a module was also defined as the module membership (MM). The module associated with a clinical feature was the one with the highest absolute MM ranking out of all the modules that were chosen.

### Protein–protein interaction network analysis

The protein–protein interaction (PPI) network was constructed using the Search Tool for the Retrieval of Interacting Genes (STRING) database (https://string-db.org), which covered almost all functional interactions between the expressed proteins, and interactions with a combined score greater than 0.4 were considered statistically significant. The results of this investigation were shown using Cytoscape software (version 3.7.0).

### Enrichment analysis

The R packages clusterProfiler and org.Hs.eg.db were used for Gene Ontology (GO) functional enrichment and Kyoto Encyclopedia of Genes and Genomes (KEGG) pathway analysis of the hub genes for all data.

### DNA extraction and quality control

Genomic DNA (gDNA) was extracted from the recently frozen tissues using the QIAmp DNA Tissue Kit (TIANGEN, Beijing, China) following the manufacturer’s instructions. The quantity and purity of the gDNA were assessed using a Qubit® 2.0 fluorometer (Thermon Fisher Scientific. Waltham, MA, USA) and a NanoDrop 2000 (Thermo Fisher Scientific, Inc.). The fragmentation status was evaluated using the Agilent 2200 TapeStation system using the Genomic DNA ScreenTape assay (Agilent Technologies, Santa Clara, CA, USA) to produce a DNA integrity number. An additional quality control step to determine the DNA integrity was performed using a multiplex PCR approach.

### Library preparation, hybridization capture, and amplification

A total of 300 ng of each gDNA sample, based on the Qubit quantification, was mechanically fragmented (duty factor, 10%; peak incident power, 175 W; cycles per burst, 200; treatment time, 240 s; bath temperature, 2–8°C) on an M220 focused ultrasonicator (Covaris, Inc.). The target DNA fragment size was 350 bp. Subsequently, 200 ng of sheared gDNA was used to perform end repair, A‑tailing, and adapter ligation with a library preparation kit (Agilent Technologies, Inc.) according to the manufacturer’s protocol. Subsequently, the libraries were captured using Agilent SureSelect XT custom 0.5–2.9M probes (Agilent Technologies, Inc.) and amplified. The captured libraries were sequenced on an Illumina NovaSeq 6000 PE150 (Illumina Inc.) for 2 × 150 paired end reads, using Cycle Sequencing v3 Reagents (Illumina).

### Bioinformatics analysis

Clean data were obtained after filtering out the adapters and reads with a proportion of N > 10%, with N being the unidentified bases in the sequencing process, using fastp (fastp 0.20.0). Low-quality bases (Phred score < 15) were excised from the 3′ ends of reads. Only sequenced samples with >80% of data with a quality score ≥ Q30 (95% of base call accuracy) were used in the analysis. Reads with length < 50 bp after excision were removed. The clean data were mapped to the human reference genome (University of California Santa Cruz ID: hg38) using the Burrows–Wheeler Alignment algorithm (BWA 0.7.17). The alignment in SAM format was converted to BAM files using SAMtools (SAMtools 1.9.0). Next, the genome analysis toolkit (GATK; v4.0.2.1) was used for sorting, duplicate marking, and base recalibration. The final BAM files were analyzed using QualiMap v.2.2.1 to provide a global overview of the data, including mapped reads, mean mapping quality, and mean coverage. The variants (single nucleotide variants (SNV) and insertion–deletion mutations (InDels)) were called for unpaired tumor sequences with 40 pooled blood samples (from healthy individuals) using the GATK mutect2 tumor-only mode (parameter: af-alleles-not-in-resource, 0.00025%), and germline mutations and contaminations were filtered out using GATK FilterMutectCalls (parameter: max-germline-posterior, 0.995). Somatic variants were annotated using the ANNOVAR software tool.

The following filter conditions were used to identify candidate somatic alterations: i) all variations with COSMIC evidence (http://cancer.sanger.ac.uk/cosmic) were retained; and ii) SNV acquisition conditions: (1) tumor samples require at least 10× coverage; (2) at least 5× coverage supports mutant alleles in tumor DNA; (3) mutant allele frequency (AF) ≥ 0.05; and (4) genomic locations with mutant allele frequencies greater than 0.1% in the Thousand Genomes Project and Exome Aggregation Consortium (ExAC) were filtered out (AF ≥ 0.001).

### Statistical analysis

The mutational landscape across the cohort was created using the maftools package in R software (R 4.3.0, R Core Team; https://www.R-Project.org). A cut-off value of an adjusted P-value (p.adjust) < 0.05 was used to identify significantly enriched terms.

## Results

### Weighted co-expression network construction and identification of key modules

**Figure 1:**
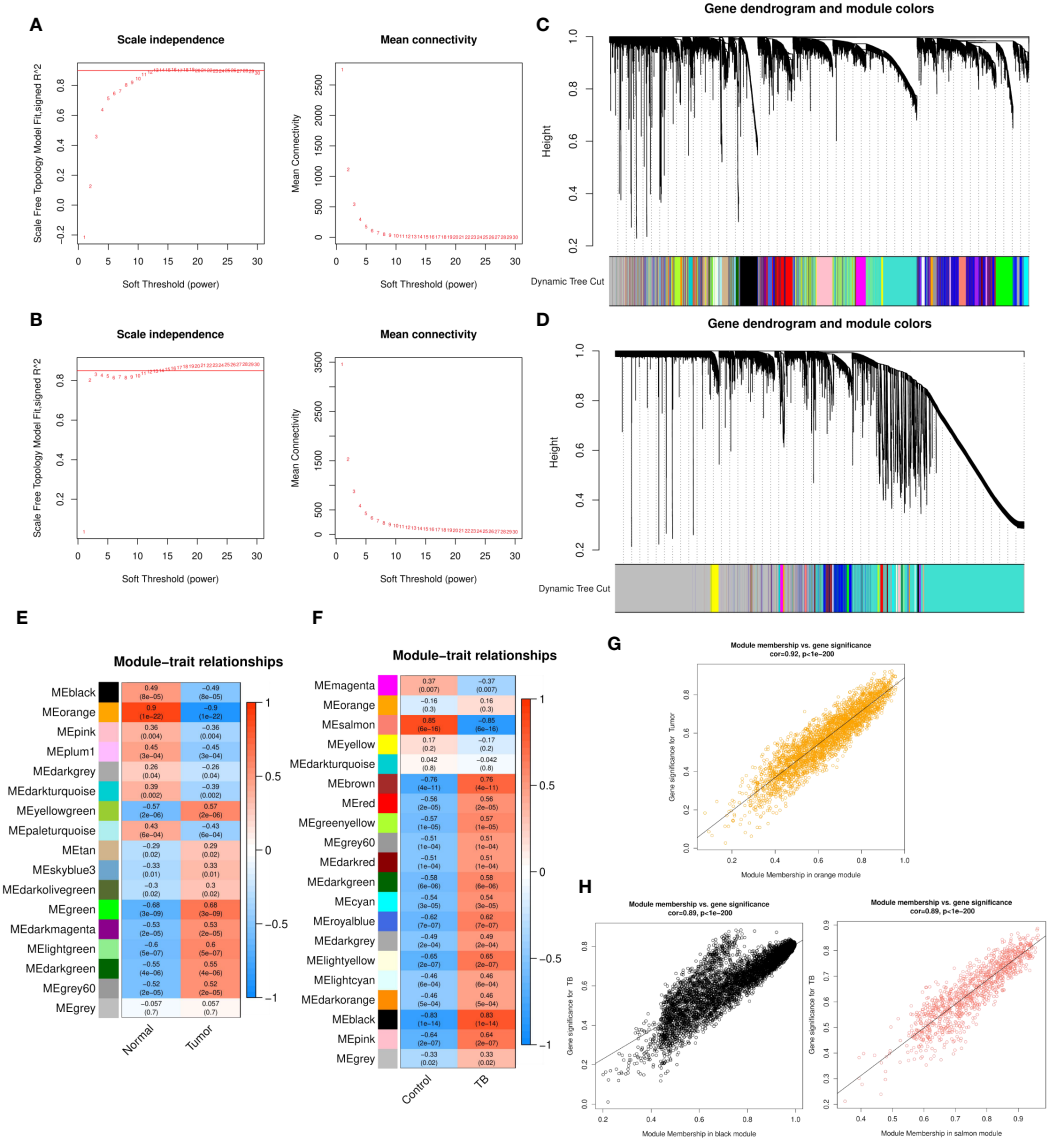
**(A, B)** Soft threshold powers (β) of 12 and 12 were selected based on the scale-free topology criterion for GSE43458 **(A)** and GSE83456 **(B)**, respectively. **(C, D)** Clustering dendrograms showing the clustering of genes with similar expression patterns into co-expression modules in GSE43458 **(C)** and GSE83456 **(D)**. The gray module indicates genes that were not assigned to any module. **(E, F)** Module–trait relationships revealing the correlations between each gene module eigengene and phenotype in GSE43458 **(E)** and GSE83456 **(F)**. **(G, H)** Scatter plots of the MM and GS of each gene in the orange module of GSE43458, showing a negative correlation with the LC proportion **(G)**, and the black and salmon modules of GSE83456 showing a positive and negative correlation, respectively, with the TB proportion **(H)**. Horizontal axis, correlation between gene and co-expression module; vertical axis, correlation between gene and phenotype. LC, lung cancer; TB, tuberculosis; MM, module membership; GS, gene significance.

WGCNA analysis was performed for both GSE43458 and GSE83456. To construct a scale-free topology network, soft threshold powers (β) of 12 in GSE43458 (scale-free R2 = 0.80) (**Figure 1A**) and 12 in GSE83456 (scale-free R2 = 0.80) (**Figure 1B**) were estimated. For GSE43458, the hierarchical clustering tree revealed that 17 co-expression modules were clustered (**Figure 1C**), and the orange module was negatively correlated with the LC proportion (Cor = 0.9, P = 1×10−22) (**Figure 1E**). For GSE83456, dynamic hybrid cutting clustered 20 co-expression models (**Figure 1D**), with the black module having the strongest positive correlation with the TB proportion (Cor = 0.83, P = 1×10−14), and the salmon module showing the strongest negative correlation (Cor = 0.85, P = 6×10−16) (**Figure 1F**). In the orange module, scatter plots showed strong negative correlations between MM and GS for LC (Cor = 0.92, P = 1×10−200) (**Figure 1G**); strong positive correlations were also observed between MM and GS for TB in the black module (Cor = 0.89, P = 1×10−200), as well as large negative correlations between MM and GS in the salmon module (Cor = 0.89, P = 1×10−200) (**Figure 1H**). Hence, these three modules were selected for in-depth investigation. A total of 5157 and 1042 genes were incorporated in the black and salmon modules, respectively, while the orange module contained 2743 genes.

### Differentially expressed genes of GSE42834

**Figure 2:**
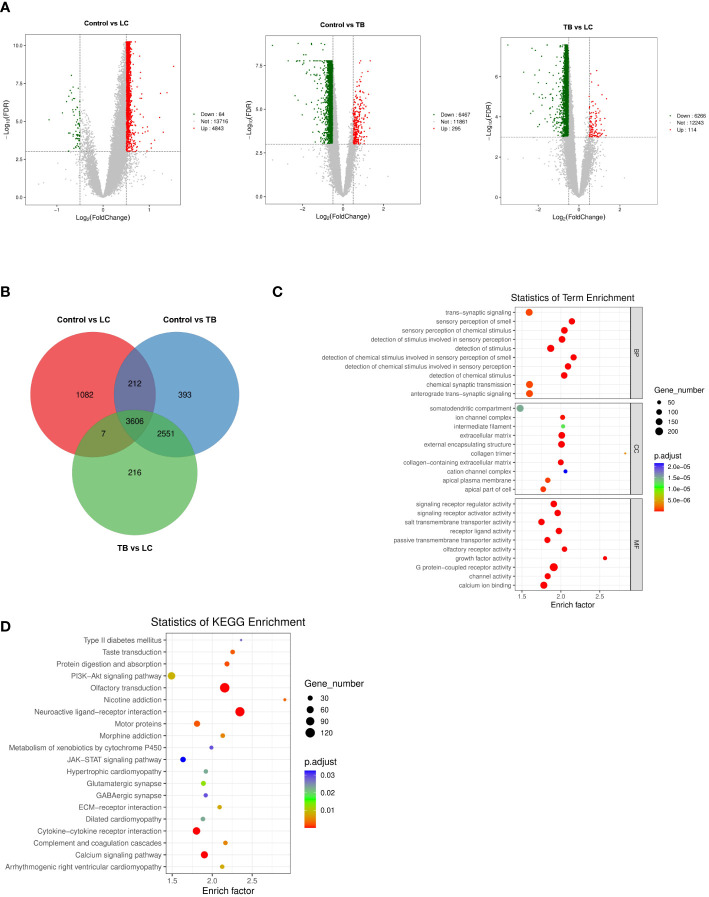
**(A)** Volcano plot visualizing DEGs in GSE42834 (20 control, 8 lung cancer, and 19 pulmonary tuberculosis samples). **(B)** Identification of common genes between the DEGs in control, lung cancer, and pulmonary tuberculosis by overlap. **(C, D)** GO analyses of the enriched BP, CC, and MF terms **(C)** and KEGG pathway analysis **(D)** of the 3606 genes. GO, Gene Ontology; KEGG, Kyoto Encyclopedia of Genes and Genomes; BP, biological process; CC, cellular component; MF, molecular function.

To identify differential genes in LC, TB, and TB patients with LC, we analyzed the DEGs in three modules using another dataset, GSE42834 from the GEO database. We set the cut-off as |log2FC| > 0.5 and q-value < 0.001 to screen DEGs from GSE42834. **Figure 2A** shows a volcano plot of the DEGs. We overlapped the DEGs and the genes in the three modules (LC vs control, LC vs TB, and TB vs control) by Venn diagram and found that 3606 common genes were present in all three modules (**Figure 2B**). **Figures 2C, D** demonstrate the GO and KEGG analyses of these 3606 genes. Extracellular matrix was the most enriched cellular component (CC) term, G protein-coupled receptor activity was the most enriched molecular function (MF) term, and neuroactive ligand–receptor interaction was the most enriched KEGG pathway.

### Functional analyses of hub genes

**Figure 3:**
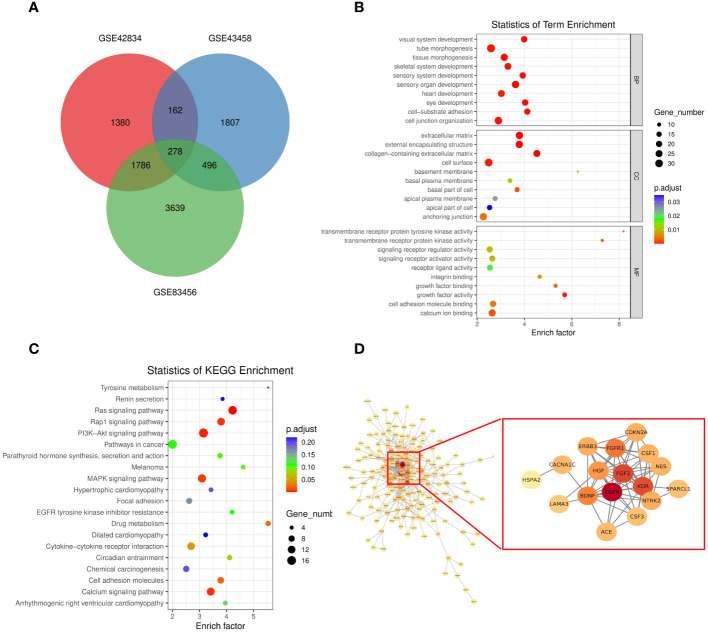
**(A)** Venn diagram showing the intersection between the orange (GSE43458), black (GSE83456), and salmon (GSE83456) module genes and the GSE42834 DEGs. **(B, C)** GO analyses of the enriched BP, CC, and MF terms **(B)** and KEGG pathway analysis **(C)** of the 278 genes. **(D)** Three-dimensional network of the 278 genes. GO, Gene Ontology; KEGG, Kyoto Encyclopedia of Genes and Genomes; BP, biological process; CC, cellular component; MF, molecular function.

To assess the function of the hub genes, we extracted the common genes derived from WGCNA and DEGs. As shown in **Figure 3A**, 278 common genes at the intersection of the three hub gene sets were detected and visualized via a Venn diagram. Subsequently, we performed GO and KEGG analyses on these 278 genes. Cell–substrate adhesion was the major enriched biological process (BP) term, and collagen-containing extracellular matrix and growth factor activity were the major enriched CC and MF terms, respectively (**Figure 3B**). The RAS and PI3K–AKT signaling pathways were the main enriched KEGG pathways (**Figure 3C**). The PPI network was constructed with 278 genes. The EGFR pathway is an oncogenic pathway in human non-small cell lung cancer (NSCLC), which also affects the levels of some pathway-related binding proteins or downstream activities (14, 15). Pathway analyses further revealed that the levels of some EGFR-related genes were altered, suggesting that EGFR might serve as a regulatory signal node in the development of TB-associated lung cancer (**Figure 3D**).

### Recurrently mutated genes in TB and LC with and without TB

**Figure 4:**
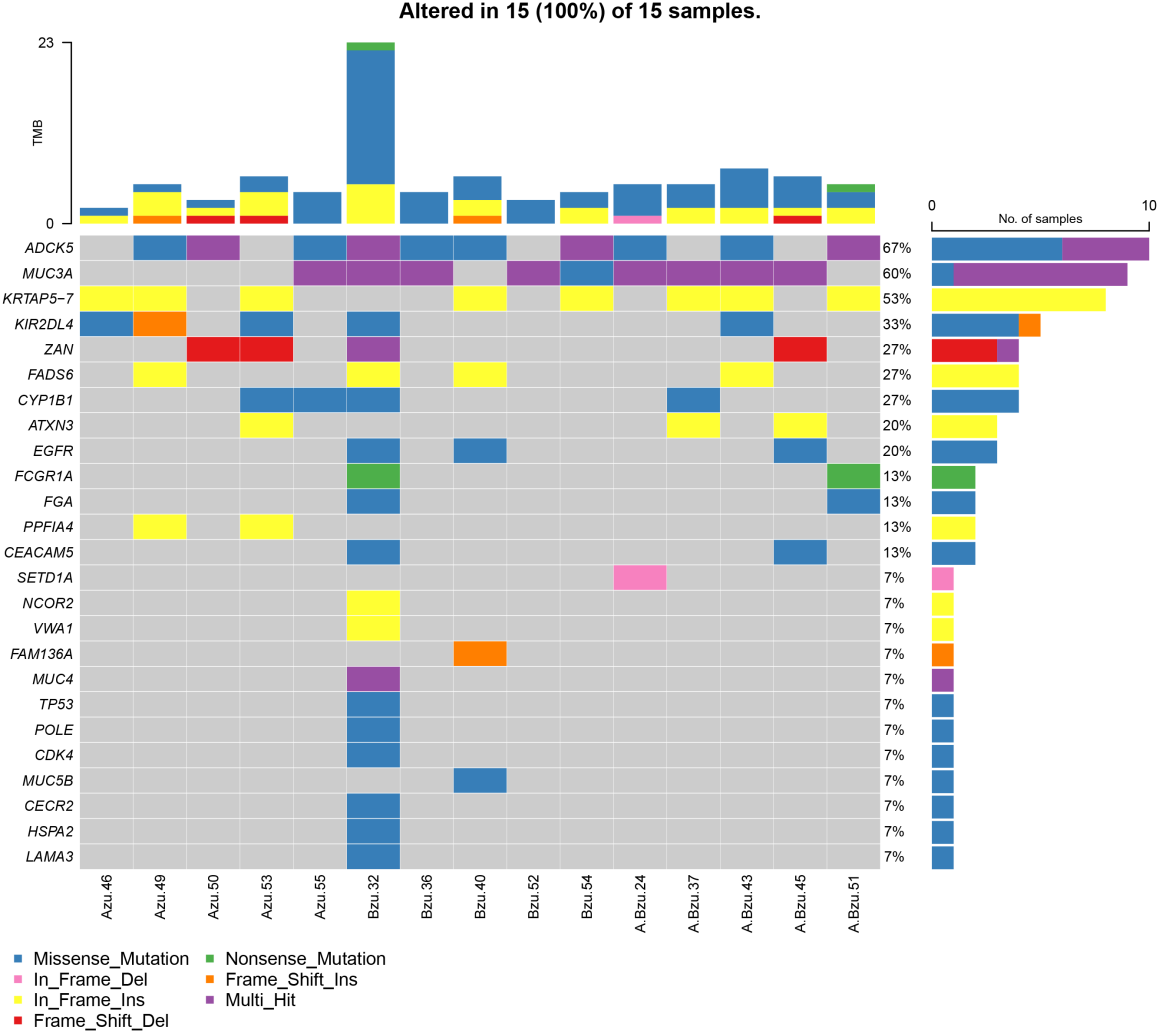
The mutational landscape of 15 patients with LC, TB, and LC+TB was determined using whole-exome sequencing. Azu, TB; Bzu, LC; A.Bzu, TB+LC; *ADCK5*, AarF domain containing kinase 5; *MUC3A*, mucin-3A; *KRTAP5–7*, keratin-associated protein 5–7; *KIR2DL4*, killer cell immunoglobulin-like receptor 2DL4; *ZAN*, zonadhesin; *FADS6*, fatty acid desaturase 6; *CYP1B1*, cytochrome P450 family 1 subfamily B member 1; *ATXN3*, ataxin-3; *EGFR*, epidermal growth factor receptor; *FCGR1A*, high affinity immunoglobulin gamma Fc receptor I; *FGA*, fibrinogen alpha chain; *PPFIA4*, liprin-alpha-4; *CEACAM5*, carcinoembryonic antigen-related cell adhesion molecule 5; *SETD1A*, histone-lysine N-methyltransferase SETD1A; *NCOR2*, nuclear receptor corepressor 2; *VWA1*, von Willebrand factor A domain-containing protein 1; *FAM136A*, family with sequence similarity 136, member A gene; *MUC4*, mucin-4; *TP53*, tumor protein p53; *MUC5B*, mucin-5B; *CECR2*, cat eye syndrome chromosome region candidate 2; *HSPA2*, heat shock protein A2; *LAMA3*, laminin subunit alpha 3.

To further investigate the role of EGFR in lung cancer and pulmonary TB, we performed exon sequencing on samples from lung cancer, lung cancer associated with pulmonary tuberculosis, and pulmonary tuberculosis patients. **Supplementary Figure S1** presents a summary of the MAF files (**Supplementary Table S6**) for the 15 patients. Totally, 2094 meaningful variations in 497 genes were identified. Totally, 2094 meaningful variations in 497 genes were identified. A waterfall plot depicts 25 of the genes containing indel mutations (**Figure 4**). For the SNVs, T > C was the most frequent SNV class. The median number of variants identified in the 15 samples was 5 (range, 1–24).

**Figure 4** presents the mutational profile of the 15 patients with LC and pulmonary TB, including 25 mutated genes, organized by the TB, LC, and TB+LC groups. The mutated gene with the highest frequency was *ADCK5* (67%). *EGFR*, one of the most frequently mutated genes in lung cancer, had a mutation rate of 20%, similar to the previously reported *EGFR* mutation rate of 5%–30% in LC (16). The other recurrently altered genes were mucin-3A (*MUC3A*; 60%), keratin-associated protein 5–7 (*KRTAP5–7*; 53%), killer cell immunoglobulin-like receptor 2DL4 (*KIR2DL4*; 33%), zonadhesin (*ZAN*; 27%), fatty acid desaturase 6 (*FADS6*; 27%), cytochrome P450 family 1 subfamily B member 1 (*CYP1B1*; 27%), and ataxin-3 (*ATXN3*; 20%). **Supplementary Figure S2** illustrates the frequency of mutations and the resulting protein structure.

### KEGG signaling pathway enrichment analysis of all somatically mutated genes

**Figure 5:**
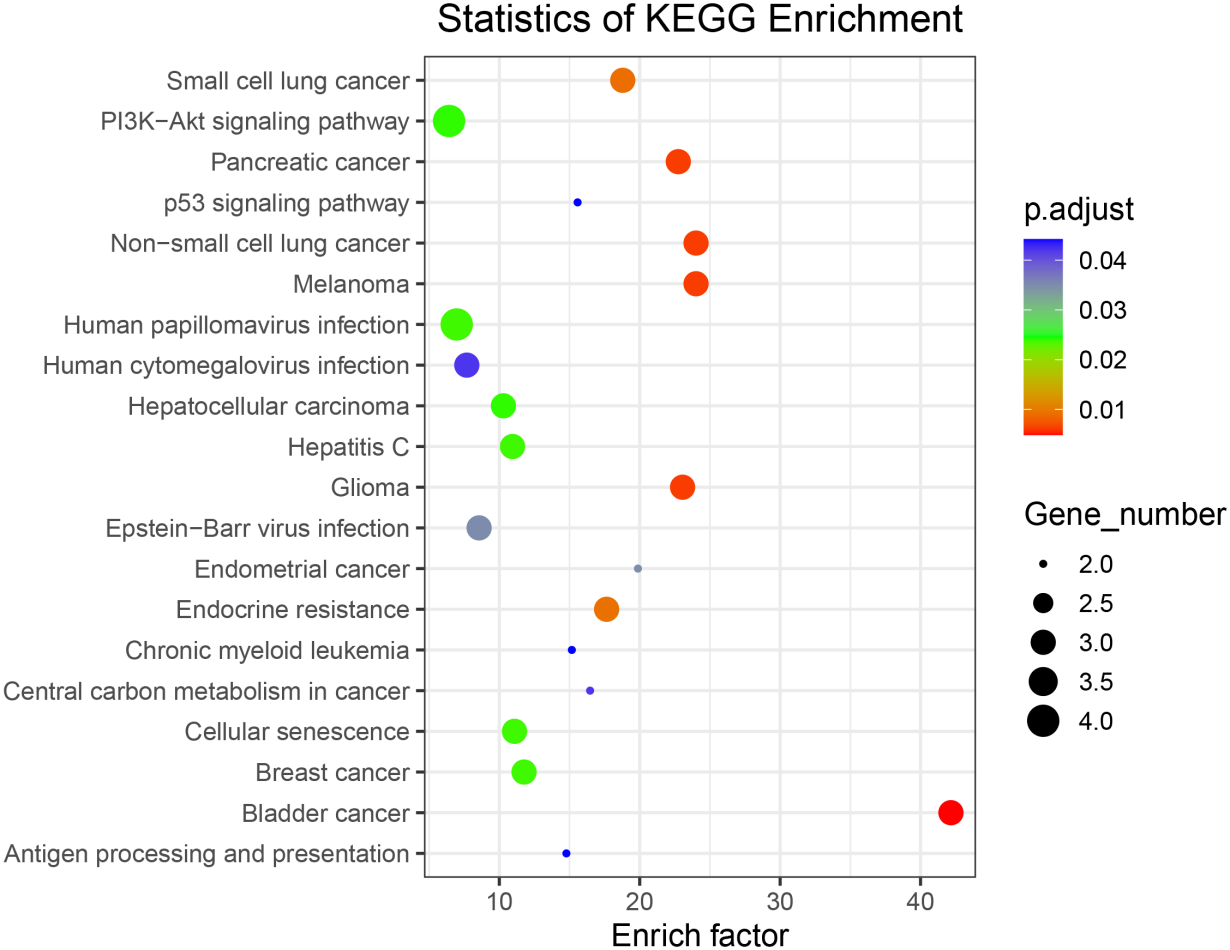
Kyoto Encyclopedia of Genes and Genomes signaling pathway enrichment analysis of all somatically mutated genes.

To further investigate the biological functions of the mutated genes, KEGG signaling pathway enrichment analyses were performed. **Supplementary Table S7** shows enriched signaling pathways associated with the mutated genes. The results of the KEGG signaling pathway analysis are presented in **Figure 5**, showing that the genes are involved in the PI3K–AKT signaling pathway and non-small cell lung cancer, which is consistent with our previous analysis.

### Correlation analysis between key genes and EGFR

**Figure 6:**
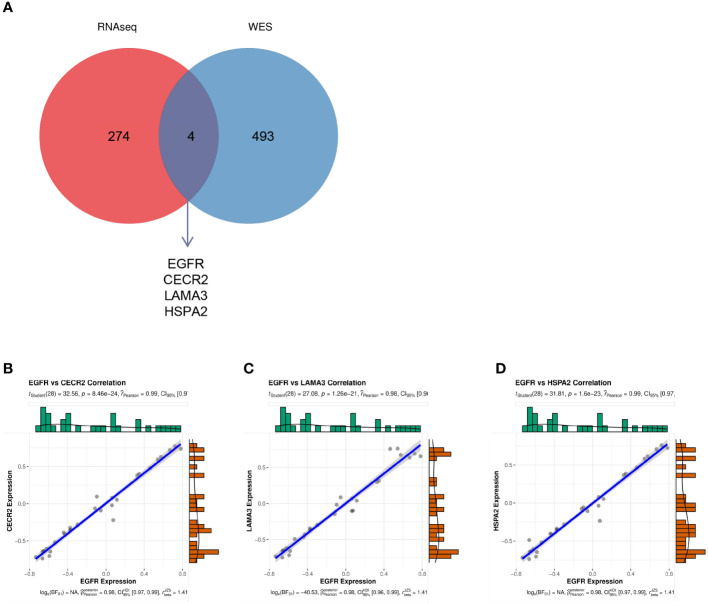
**(A)** The intersection between the 278 hub genes and the mutated genes shown in a Venn diagram. **(B–D)** The correlation between *EGFR* and *CECR2* **(B)**, *LAMA3* **(C)**, and *HSPA2* **(D)**.

EGFR had the highest degree in the aforementioned PPI, with a mutation rate of 20% in lung cancer. This implies that EGFR is involved in the progression of lung cancer. Thus, we investigated the relationship between EGFR and other genes. The Venn diagram in **Figure 6A** depicts the genes common to both the 278 hub genes and all mutated genes, including *EGFR*, *HSPA2*, *CECR2*, and *LAMA3* (**Figure 6A**). To investigate the effects of *EGFR* expression on *HSPA2*, *CECR2*, and *LAMA3*, we performed the CIBERSORT algorithm on 15 tumor samples to calculate the expression of *EGFR* and the three key genes in each sample. As shown in **Figures 6B–D**, *CECR2*, *LAMA3*, and *HSPA2* were positively correlated with *EGFR* expression.

## Discussion

Lung cancer is the most dangerous of the common malignant tumors in China, causing the most cancer deaths each year (17). Tuberculosis is an infectious illness of the lungs caused by *Mycobacterium tuberculosis*, and tuberculosis of the lungs raises the risk of a patient getting lung cancer by causing inflammatory irritation that leads to persistent irritation of the lungs (18, 19). Younger patients show a significantly higher association between TB and lung cancer (20). Several studies have shown that LC in TB patients have lower survival rates than non-TB patients (21, 22). A study of 6934 patients among patients with primary cancer and TB infection demonstrated that TB is a risk for facilitating primary cancer to metastasize to the lung (23). Delayed diagnosis and treatment of TB increases the chance of patient complications and mortality and enhances TB transmission in the population (24). Therefore, it is of practical significance to explore the mechanism of the association between TB and lung cancer development and provide new targets for clinical examination and future targeted therapy of lung cancer patients.

In this study, WGCNA was performed by extracting co-expression networks of grouped genes from a large expression dataset. Among the 37 modules, we found that the orange, black, and salmon modules were most significantly related to LC or TB. We analyzed the GSE42834 dataset, which includes LC, TB, and control groups, to find 3606 DEGs. The confluence of these differential genes with the genes from the three WGCNA modules resulted in 278 genes for which we determined the PPI network, showing that *EGFR* and related genes are highly correlated with TB and LC. KEGG pathway analysis revealed that the hub genes were primarily enriched in pathways related to growth, survival, and metabolism of cancer cells, such as the RAS and PI3K–AKT signaling pathways. The PI3K–AKT signaling pathway is dysregulated in almost all cancers due to gene amplification (25). Studies suggest that in patients with an *EGFR* mutation, the AKT/mTOR pathway is constitutively activated in 67% of cases (26, 27). RAS signaling is a major nexus controlling essential cell parameters, including proliferation, survival, and migration, utilizing downstream effectors such as the PI3K–AKT signaling pathway (28, 29).

Next, we examined the somatic mutation patterns of 15 individuals to acquire a better understanding of the progression from TB to LC. In addition to somatic alterations in previously known LC-associated genes, such as *EGFR*, *ADCK5*, *MUC3A*, and *KIR2DL4 (* 16, 30–33), we identified mutations in new genes, such as *CECR2*, *LAMA3*, *FADS6*, *CYP1B1*, and *ATXN3*. Interestingly, we found that four (*EGFR*, *CECR2*, *LAMA3*, and *HSPA2*) of the 278 genes obtained in the three GEO datasets were mutated in all 15 patients. *EGFR* had a mutation rate of 20%, similar to the previously reported *EGFR* mutation rate (5%–30%) in LC (16). The epidermal growth factor receptor (*EGFR*) gene encodes signaling proteins crucial for cell proliferation and survival, and *EGFR* mutations are major driver mutations occurring in lung adenocarcinomas (16, 34, 35). The incidence of LC *EGFR* mutations was found to be higher in East Asian countries, as was the prevalence of TB infection (2, 34). Studies have examined the expression of *CECR2* in breast cancer and found that it regulates the tumor immune microenvironment to promote breast cancer metastasis (29). However, there have been no reports to date implicating *CECR2* in LC. Reducing the methylation of the *LAMA3* promoter inhibits the proliferation, invasion, migration, and drug resistance of lung adenocarcinoma cells (36). The data reported show that *HSPA2* does not promote a malignant NSCLC phenotype. HSPA2-deficient keratinocytes show accelerated differentiation in a reconstituted human epidermis model (37–39). These four genes may be key proteins that predict the development of LC in TB. Additionally, the altered genes were discovered to be highly enriched in the PI3K–AKT signaling pathway, consistent with other previous studies (40–42). Nevertheless, further research is required to fully explore their roles in TB and LC.

We acknowledge that there were some limitations and shortcomings of this study. First, this study was mainly focused on data mining and data analysis, which are based on methodology. Clinical information available in public databases is limited, and contaminated tissues and biases in sequencing may lead to biased results in WGCNA. In addition, after obtaining the hub gene, the association with the tumor microenvironment should also be analyzed and further verified by experiments (43, 44). Second, the single method of whole-exon sequencing used, and the minimal sample size, may have an impact on the accuracy of the results. Our future research will include large sample sizes, analyzed by different methods.

## Conclusion

We applied WGCNA and WES to explore the development of LC, and determined a mutational profile of 15 patients by WES. This identified four genes (*EGFR*, *CECR2*, *LAMA3*, and *HSPA2*) that play an important role in LC tumorigenesis. Furthermore, the present study confirms *EGFR* mutations in LC and verifies the enrichment of gene alterations in the PI3K–AKT signaling pathway in a small cohort of Chinese patients with LC. These results may shed light on opportunities for diagnosis and personalized treatment of TB with LC.

## Data availability statement

The datasets used and/or analyzed during the current study are available from the GEO database (ID: GSE43458, https://www.ncbi.nlm.nih.gov/geo/query/acc.cgi?acc=GSE43458; GSE83456, https://www.ncbi.nlm.nih.gov/geo/query/acc.cgi?acc=GSE83456; GSE42834, https://www.ncbi.nlm.nih.gov/geo/query/acc.cgi?acc=GSE42834. The WES datasets used and/or analyzed during the current study are available from the BioProject database (BioProject ID: PRJNA1080061; https://www.ncbi.nlm.nih.gov/bioproject/PRJNA1080061).

## Ethics statement

The studies involving humans were approved by Ethics Committees of The Xinjiang Uygur Autonomous Region Chest Hospital (approval number 2021BAT011). The studies were conducted in accordance with the local legislation and institutional requirements. The participants provided their written informed consent to participate in this study. Written informed consent was obtained from the individual(s) for the publication of any potentially identifiable images or data included in this article.

## Author contributions

MA: Conceptualization, Writing – original draft. ZL: Investigation, Resources, Visualization, Writing – review & editing. BA: Data curation, Methodology, Project administration, Writing – review & editing.

## Supplementary material

SUPPLEMENTARY FIGURE S1 Summary and visualization of maf files using the maftools package in R software. **(A)** Variant classification. **(B)** Variant type. **(C)** SNV class. **(D)** The count of variants per sample. **(E)** Variant classification summary. **(F)** Top 30 mutated genes. The colors of variant classification in subfigure D, E and F are in accordance with subfigure A. Del, deletion; Ins, insertion; SNP, single nucleotide polymorphism; SNV, single nucleotide variant.

SUPPLEMENTARY FIGURE S2 Proportion of all mutated genes.

## References

[1] Siegel RL Miller KD Wagle NS Jemal A. Cancer statistics, 2023. CA: Cancer J Clin. (2023) 73:17–48. 36633525 10.3322/caac.21763

[2] Luo YH Wu CH Wu WS Huang CY Su WJ Tsai CM. Association between tumor epidermal growth factor receptor mutation and pulmonary tuberculosis in patients with adenocarcinoma of the lungs. J Thorac oncology: Off Publ Int Assoc Study Lung Cancer. (2012) 7:299–305. 10.1097/JTO.0b013e31823c588d 22173705

[3] Yu YH Liao CC Hsu WH Chen HJ Liao WC Muo CH. Increased lung cancer risk among patients with pulmonary tuberculosis: a population cohort study. J Thorac oncology: Off Publ Int Assoc Study Lung Cancer. (2011) 6:32–7. 10.1097/JTO.0b013e3181fb4fcc 21150470

[4] Keikha M Esfahani BN. The relationship between tuberculosis and lung cancer. Advanced Biomed Res. (2018) 7:58. 10.4103/abr.abr_182_17 PMC5887688 29657943

[5] Hong S Mok Y Jeon C Jee SH Samet JM. Tuberculosis, smoking and risk for lung cancer incidence and mortality. Int J cancer. (2016) 139:2447–55. 10.1002/ijc.30384 27521774

[6] Wu CY Hu HY Pu CY Huang N Shen HC Li CP. Pulmonary tuberculosis increases the risk of lung cancer: a population-based cohort study. Cancer. (2011) 117:618–24. 10.1002/cncr.25616 20886634

[7] Everatt R Kuzmickiene I Davidaviciene E Cicenas S. Incidence of lung cancer among patients with tuberculosis: a nationwide cohort study in Lithuania. Int J tuberculosis Lung disease: Off J Int Union against Tuberculosis Lung Disease. (2016) 20:757–63. 10.5588/ijtld.15.0783 27155178

[8] Bhowmik S Mohanto NC Sarker D Sorove AA. Incidence and risk of lung cancer in tuberculosis patients, and vice versa: A literature review of the last decade. BioMed Res Int. (2022) 2022:1702819. 36578803 10.1155/2022/1702819 PMC9792248

[9] Zifodya JS Crothers K. Tuberculosis, chronic obstructive lung disease, and lung cancer: the holey upper lobe trinity? Ann Am Thorac Soc. (2022) 19:540–2. 10.1513/AnnalsATS.202201-009ED PMC8996276 35363131

[10] Hart JL Turnbull AE Oppenheim IM Courtright KR. Family-centered care during the COVID-19 era. J Pain symptom management. (2020) 60:e93–e7. 10.1016/j.jpainsymman.2020.04.017 PMC7175858 32333961

[11] Hart JL Taylor SP. Family presence for critically ill patients during a pandemic. Chest. (2021) 160:549–57. 10.1016/j.chest.2021.05.003 PMC8105126 33971149

[12] Nanthanangkul S Promthet S Suwanrungruang K Santong C Vatanasapt P. Incidence of and risk factors for tuberculosis among cancer patients in endemic area: A regional cohort study. Asian Pacific J Cancer prevention: APJCP. (2020) 21:2715–21. 10.31557/APJCP.2020.21.9.2715 PMC7779434 32986373

[13] Cokus S Rose S Haynor D Grønbech-Jensen N Pellegrini M. Modelling the network of cell cycle transcription factors in the yeast Saccharomyces cerevisiae. BMC Bioinf. (2006) 7:381. 10.1186/1471-2105-7-381 PMC1570153 16914048

[14] Yu JJ Zhou DD Yang XX Cui B Tan FW Wang J. TRIB3-EGFR interaction promotes lung cancer progression and defines a therapeutic target. Nat Commun. (2020) 11:3660. 32694521 10.1038/s41467-020-17385-0 PMC7374170

[15] Bao Y Wang L Shi L Yun F Liu X Chen Y. Transcriptome profiling revealed multiple genes and ECM-receptor interaction pathways that may be associated with breast cancer. Cell Mol Biol letters. (2019) 24:38. 10.1186/s11658-019-0162-0 PMC6554968 31182966

[16] Bronte G Rizzo S La Paglia L Adamo V Siragusa S Ficorella C. Driver mutations and differential sensitivity to targeted therapies: a new approach to the treatment of lung adenocarcinoma. Cancer Treat Rev. (2010) 36 Suppl 3:S21–9. 10.1016/S0305-7372(10)70016-5 21129606

[17] Torre LA Siegel RL Jemal A. Lung cancer statistics. Adv Exp Med Biol. (2016) 893:1–19. 26667336 10.1007/978-3-319-24223-1_1

[18] Sharma A Sharma A Malhotra R Singh P Chakrabortty RK Mahajan S. An accurate artificial intelligence system for the detection of pulmonary and extra pulmonary Tuberculosis. Tuberculosis (Edinburgh Scotland). (2021) 131:102143. 34794086 10.1016/j.tube.2021.102143

[19] Ehrt S Schnappinger D Rhee KY. Metabolic principles of persistence and pathogenicity in Mycobacterium tuberculosis. Nat Rev Microbiol. (2018) 16:496–507. 29691481 10.1038/s41579-018-0013-4 PMC6045436

[20] Hwang SY Kim JY Lee HS Lee S Kim D Kim S. Pulmonary tuberculosis and risk of lung cancer: A systematic review and meta-analysis. J Clin Med. (2022) 11:765. 10.3390/jcm11030765 PMC8836400 35160218

[21] Abdelwahab HW Elmaria MO Abdelghany DA Akl FM Shehta M ELnagar RM. Screening of latent TB infection in patients with recently diagnosed bronchogenic carcinoma. Asian Cardiovasc Thorac Ann. (2021) 29:208–13. 10.1177/0218492320984881 33375818

[22] Kumar DS Ronald LA Romanowski K Rose C Shulha HP Cook VJ. Risk of active tuberculosis in migrants diagnosed with cancer: a retrospective cohort study in British Columbia, Canada. BMJ Open. (2021) 11:e037827. 10.1136/bmjopen-2020-037827 PMC7929860 33653739

[23] Ho LJ Yang HY Chung CH Chang WC Yang SS Sun CA. Increased risk of secondary lung cancer in patients with tuberculosis: A nationwide, population-based cohort study. PloS One. (2021) 16:e0250531. 33961650 10.1371/journal.pone.0250531 PMC8104424

[24] Santos JA Leite A Soares P Duarte R Nunes C. Delayed diagnosis of active pulmonary tuberculosis - potential risk factors for patient and healthcare delays in Portugal. BMC Public Health. (2021) 21:2178. 34837969 10.1186/s12889-021-12245-y PMC8627051

[25] Asati V Mahapatra DK Bharti SK. PI3K/Akt/mTOR and Ras/Raf/MEK/ERK signaling pathways inhibitors as anticancer agents: Structural and pharmacological perspectives. Eur J Medicinal Chem. (2016) 109:314–41. 10.1016/j.ejmech.2016.01.012 26807863

[26] Dobashi Y Koyama S Kanai Y Tetsuka K. Kinase-driven pathways of EGFR in lung carcinomas: perspectives on targeting therapy. Front Bioscience (Landmark edition). (2011) 16:1714–32. 10.2741/3815 21196258

[27] Tan AC. Targeting the PI3K/Akt/mTOR pathway in non-small cell lung cancer (NSCLC). Thorac Cancer. (2020) 11:511–8. 10.1111/1759-7714.13328 PMC7049515 31989769

[28] Cox AD Der CJ. Ras history: The saga continues. Small GTPases. (2010) 1:2–27. 21686117 10.4161/sgtp.1.1.12178 PMC3109476

[29] Sittewelle M Kappès V Zhou C Lécuyer D Monsoro-Burq AH. PFKFB4 interacts with ICMT and activates RAS/AKT signaling-dependent cell migration in melanoma. Life Sci Alliance. (2022) 5:e202201377. 10.26508/lsa.202201377 PMC9348664 35914811

[30] Okada F Takeda M Fujii T Uchiyama T Sasaki S Matsuoka M. Clinicopathological and genetic analyses of pulmonary enteric adenocarcinoma. J Clin Pathol. (2024) 77:111–5. 10.1136/jcp-2022-208583 36456172

[31] Sun Y Sun X You C Ma S Luo Y Peng S. MUC3A promotes non-small cell lung cancer progression via activating the NFκB pathway and attenuates radiosensitivity. Int J Biol Sci. (2021) 17:2523–36. 10.7150/ijbs.59430 PMC8315024 34326691

[32] Qiu M Li G Wang P Li X Lai F Luo R. aarF domain containing kinase 5 gene promotes invasion and migration of lung cancer cells through ADCK5-SOX9-PTTG1 pathway. Exp Cell Res. (2020) 392:112002. 32277958 10.1016/j.yexcr.2020.112002

[33] Wiśniewski A Kowal A Wyrodek E Nowak I Majorczyk E Wagner M. Genetic polymorphisms and expression of HLA-G and its receptors, KIR2DL4 and LILRB1, in non-small cell lung cancer. Tissue Antigens. (2015) 85:466–75. 10.1111/tan.12561 25855135

[34] Lynch TJ Bell DW Sordella R Gurubhagavatula S Okimoto RA Brannigan BW. Activating mutations in the epidermal growth factor receptor underlying responsiveness of non-small-cell lung cancer to gefitinib. New Engl J Med. (2004) 350:2129–39. 10.1056/NEJMoa040938 15118073

[35] Jett JR Carr LL. Targeted therapy for non-small cell lung cancer. Am J Respir Crit Care Med. (2013) 188:907–12. 10.1164/rccm.201301-0189PP 23721055

[36] Xu SF Zheng Y Zhang L Wang P Niu CM Wu T. Long non-coding RNA LINC00628 interacts epigenetically with the LAMA3 promoter and contributes to lung adenocarcinoma. Mol Ther Nucleic Acids. (2019) 18:166–82. 10.1016/j.omtn.2019.08.005 PMC6796683 31557618

[37] Zhang M Liu ZZ Aoshima K Cai WL Sun H Xu T. CECR2 drives breast cancer metastasis by promoting NF-κB signaling and macrophage-mediated immune suppression. Sci Trans Med. (2022) 14:eabf5473. 10.1126/scitranslmed.abf5473 PMC9003667 35108062

[38] Raiford KL Park J Lin KW Fang S Crews AL Adler KB. Mucin granule-associated proteins in human bronchial epithelial cells: the airway goblet cell “granulome”. Respir Res. (2011) 12:118. 21896166 10.1186/1465-9921-12-118 PMC3184067

[39] Fang S Crews AL Chen W Park J Yin Q Ren XR. MARCKS and HSP70 interactions regulate mucin secretion by human airway epithelial cells *in vitro* . Am J Physiol Lung Cell Mol Physiol. (2013) 304:L511–8. 10.1152/ajplung.00337.2012 PMC3625989 23377348

[40] Tian X Gu T Lee MH Dong Z. Challenge and countermeasures for EGFR targeted therapy in non-small cell lung cancer. Biochim Biophys Acta Rev Cancer. (2022) 1877:188645. 34793897 10.1016/j.bbcan.2021.188645

[41] Wu S Luo M To KKW Zhang J Su C Zhang H. Intercellular transfer of exosomal wild type EGFR triggers osimertinib resistance in non-small cell lung cancer. Mol Cancer. (2021) 20:17. 33461557 10.1186/s12943-021-01307-9 PMC7812728

[42] Kharbanda A Walter DM Gudiel AA Schek N Feldser DM Witze ES. Blocking EGFR palmitoylation suppresses PI3K signaling and mutant KRAS lung tumorigenesis. Sci Signaling. (2020) 13:eaax2364. 10.1126/scisignal.aax2364 PMC7310254 32127496

[43] Dai S Cao T Shen H Zong X Gu W Li H. Landscape of molecular crosstalk between SARS-CoV-2 infection and cardiovascular diseases: emphasis on mitochondrial dysfunction and immune-inflammation. J Trans Med. (2023) 21:915. 10.1186/s12967-023-04787-z PMC10725609 38104081

[44] Hu Y Zeng N Ge Y Wang D Qin X Zhang W. Identification of the shared gene signatures and biological mechanism in type 2 diabetes and pancreatic cancer. Front Endocrinology. (2022) 13:847760. 10.3389/fendo.2022.847760 PMC9010232 35432196

