# Supplementary figures and images for: Weighted gene co-expression network analysis and whole genome sequencing identify potential lung cancer biomarkers
Source: Front Oncol. 2024 May 24;14:1355527. doi: 10.3389/fonc.2024.1355527 (PMC11157001; doi:10.3389/fonc.2024.1355527)

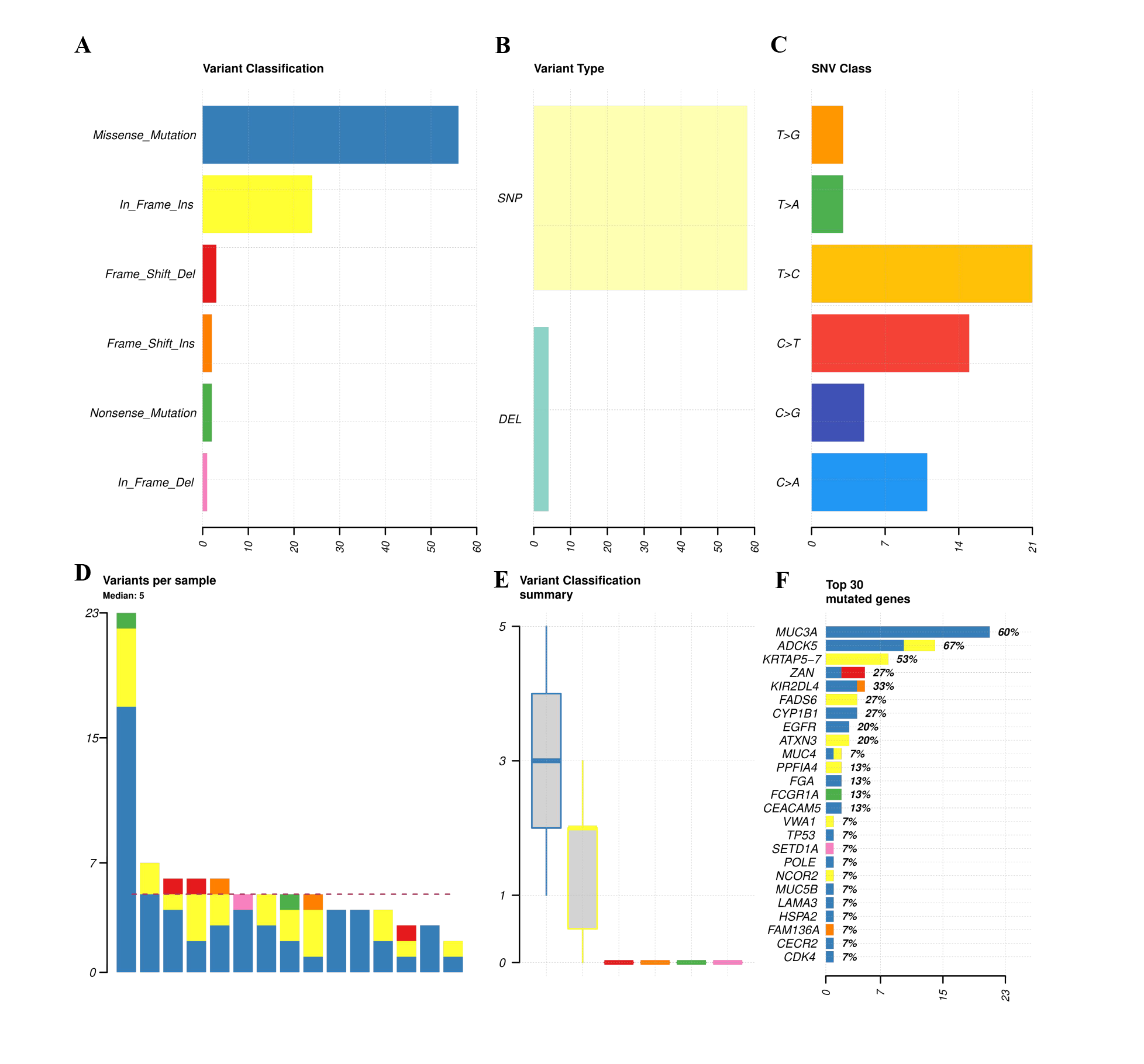

Supplement: SUPPLEMENTARY FIGURE S1 — Summary and visualization of maf files using the maftools package in R software. (A) Variant classification. (B) Variant type. (C) SNV class. (D) The count of variants per sample. (E) Variant classification summary. (F) Top 30 mutated genes. The colors of variant classification in subfigure D, E and F are in accordance with subfigure A. Del, deletion; Ins, insertion; SNP, single nucleotide polymorphism; SNV, single nucleotide variant. [file Image_1.tif]

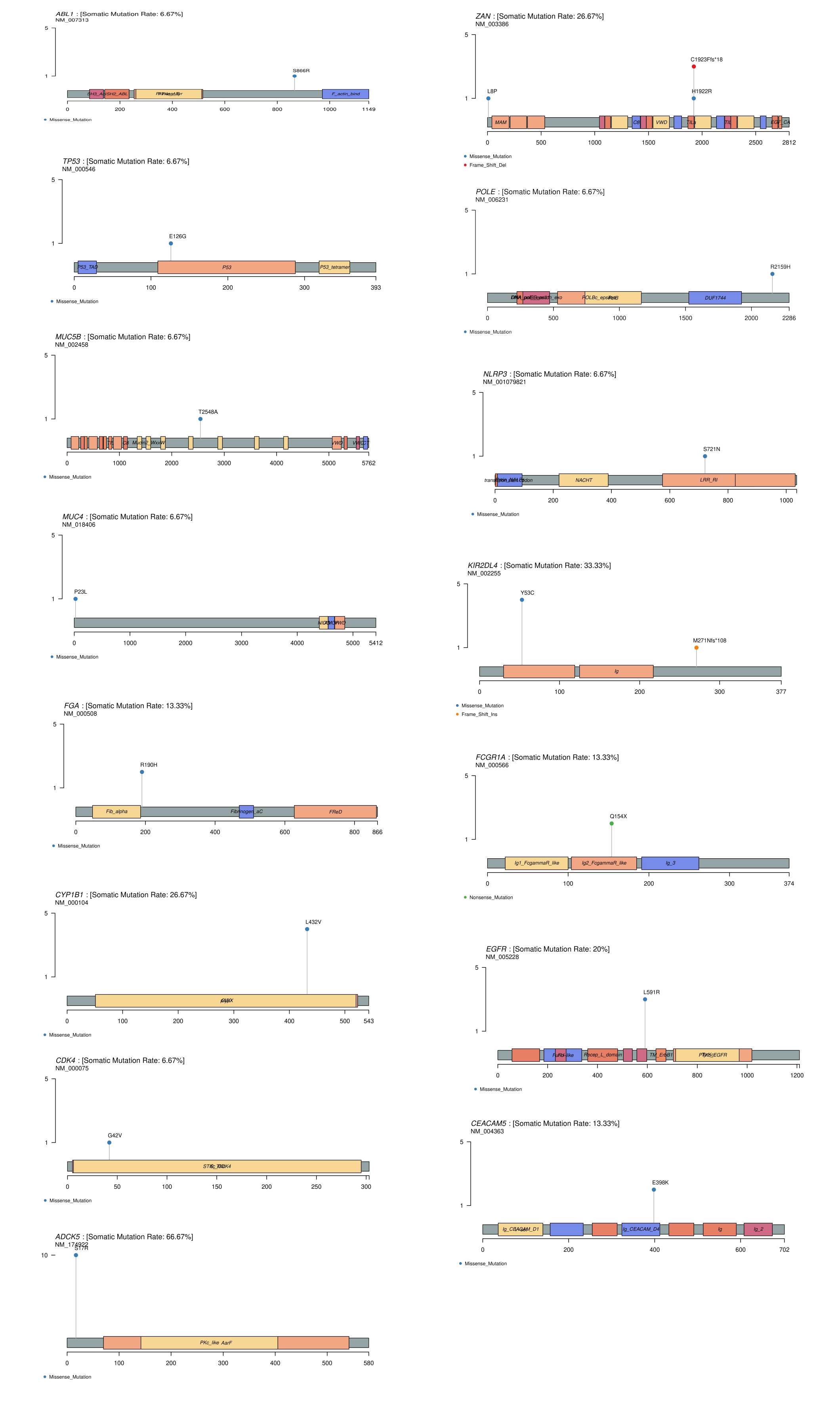

Supplement: SUPPLEMENTARY FIGURE S2 — Proportion of all mutated genes. [file Image_2.tif]
